# Enhancing the Photocatalytic Activity of SnO_2_-TiO_2_ and ZnO-TiO_2_ Tandem Structures Toward Indoor Air Decontamination

**DOI:** 10.3389/fchem.2020.583270

**Published:** 2020-11-25

**Authors:** Alexandru Enesca

**Affiliations:** Product Design, Mechatronics and Environmental Department, Transilvania University of Brasov, Braşov, Romania

**Keywords:** air decontamination, acetaldehyde, photocatalysis, metal oxides, tandem structures

## Abstract

ZnO-TiO_2_ and SnO_2_-TiO_2_ tandem structures were developed using the doctor blade technique. It was found that by employing organic hydrophilic and hydrophobic as additives into the precursor it is possible to tailor the film density and morphology with direct consequences on the photocatalytic activity of the tandem structures. The highest photocatalytic efficiency corresponds to ZnO-TiO_2_ and can reach 74.04% photocatalytic efficiency toward acetaldehyde when a hydrophilic additive is used and 70.93% when a hydrophobic additive is employed. The snO_2_-TiO_2_ tandem structure presents lower photocatalytic properties (61.35 % when the hydrophilic additive is used) with a constant rate reaction of 0.07771 min^−1^.

## Introduction

The concentration of volatile organic compounds (VOCs) in the atmosphere varies over a wide range of values and depends on several factors such as the local geography, industrialization, proximity to the emission source, climate, etc. (Liu et al., 2012; Han et al., 2017; Lomonaco et al., 2020). The recognition that poor air quality will affect human health and productivity was a realization and major social concern of the twentieth century (Costa et al., 2020). Most of the air decontamination studies that have been conducted to date are focused on SO_x_, NO_x_, and VOCs as the main causes of the greenhouse effect, (Xiong et al., 2019; Liu et al., 2020). Another important aspect is represented by indoor air pollution is the fact that many people usually live and work in closed spaces (Zhou et al., 2017). Acetaldehyde is a subject of interest for researchers analyzing indoor pollution (Amoatey et al., 2018; Chen et al., 2020), as it can be released for long periods by different construction materials such as polyurethane foams, adhesives, coatings, inks, and consumer products like cigarettes (Wang et al., 2016; Lin et al., 2017; Taylor et al., 2019). Direct contact with acetaldehyde irritates the skin and eyes, while short term inhalation affects the human respiratory system. Additionally, acetaldehyde can have a damaging effect on the cardiovascular system and it is suspected to be a human carcinogen (Solsona et al., 2012; Ohashi et al., 2015).

Different mono-component metal oxide photocatalyst such as ZnO (He et al., 2018; Smazna et al., 2019), TiO_2_ (Andronic et al., 2014; Castellanos et al., 2020), WO_3_ (Yang et al., 2018; Zhao et al., 2020) or SnO_2_ (Enesca et al., 2012a; Patil et al., 2018) were widely used for wastewater and air pollutant removal. Although efficient, these materials have limitations in terms of charge carrier recombination, absorption range, and photoactive crystalline structure. The use of tandem systems such as CdS/TiO_2_ (Isac et al., 2019; Wang et al., 2019), SnO_2_/ZnO (Ali et al., 2016; Xu et al., 2019b), ZnO/TiO_2_ (Meng et al., 2018; Wetchakun et al., 2019), TiO_2_/SiO_2_ (Liu et al., 2012b; Enesca et al., 2017), WO_3_/TiO_2_ (Enesca et al., 2012b; Rhaman et al., 2020), SiO_2_/ZnO (Xu et al., 2019a; Nasseh et al., 2020), or SnO_2_/TiO_2_ (Enesca et al., 2014; Huy et al., 2019) has improved photocatalytic properties by reducing charge carriers recombination rate and extending the absorbance spectrum. However, most of these studies are made in a liquid environment and cannot be directly transferred to gaseous predictions for the removal of pollutants (Jeleńska et al., 2017). The improvement of photocatalytic systems for the removal of an indoor air pollutant is required before studies can undertake large scale applications.

The present paper explores the possibility to enhance the photocatalytic activity of SnO_2_-TiO_2_ and ZnO-TiO_2_ tandem structures by tailoring the morphology. The use of hydrophilic and hydrophobic polymers as additives in the doctor blade technique could enable more accurate control of the morphology of films, with direct implications on the photocatalytic activity of the tandem structures. The removal of acetaldehyde in a gaseous environment was tested for 12 h under UV irradiation to demonstrate the ability of these structures to promote organic pollutant mineralization in confined spaces.

## Experimental

### Photocatalysts Materials

Four samples were prepared based on the following procedure:

1. Sample Sn_Ti_HL film was developed on microscopic glass (MG) substrate by doctor blade technique. The paste was obtained by mixing SnO_2_ (AlfaAesar, 99.98%) and TiO_2_ Degussa P25 powders (mass ratio 5:1) into solutions containing ethanol, sodium maleat-methyl metacrylate hydrophilic (HL) additive, triton X100 in a volumetric ratio 10:2:1.
2. Sample Sn_Ti_HB film was deposed on MG substrate by doctor blade technique. The paste was obtained by mixing SnO_2_ and TiO_2_ Degussa P25 powders (mass ratio 5:1) into solutions containing ethanol, sodium maleat-vinyl acetate hydrophobic (HB) additive, triton X100 in a volumetric ratio 10:2:1.
3. Sample Zn_Ti_HL film was developed on MG substrate by doctor blade technique. The paste was obtained by mixing ZnO (AlfaAesar, 99.98%) and TiO_2_ Degussa P25 powders (mass ratio 5:1) into solutions containing ethanol, HL additive, triton X100 in a volumetric ratio 10:2:1.
4. Sample Zn_Ti_HB film was deposed on MB substrate by doctor blade technique. The paste was obtained by mixing ZnO and TiO_2_ Degussa P25 powders (mass ratio 5:1) into solutions containing ethanol, HB additive, triton X100 in a volumetric ratio 10:2:1.

Previously, the substrates with identical sizes (1.5 × 2.5 cm^2^) were degreased with specific surfactants and cleaned by successive immersion in acetone and ethanol for 20 min using an ultrasound bath. After deposition, the samples were thermally treated in a reach oxygen atmosphere at 450°C for 12 h.

### Characterization Techniques

The presence of crystalline structures and composition of the tandem structures were identified using X-Ray Diffraction analysis using a Rigaku Miniflex X-Ray diffractometer containing a Cu K_α_ source (I = 1.54 Å, 40 kV, 100 mA). The morphology investigations were done by field emission scanning electron microscopy (FESEM, SU8010), operated at an accelerated voltage of 25 kV. The optical properties (absorbance and reflectance) necessary to evaluate the film thickness and the band gap were investigated using a UV-Vis spectrometer (PerkinElmer Lambda 25 UV/Vis).

### Photocatalytic Experiments

The photocatalytic activity vs. acetaldehyde was tested for each tandem structure in a quartz air-proof box. An amount corresponding to 150 ppm of acetaldehyde was injected into the test box. The boxes had been previously filled with dry air for 30 min. Before the experiment, the tandem structures were kept for 24 h under UV irradiation (0.6 mW/cm^2^). The photocatalytic experiments consist of two steps: (1) the samples were kept in dark for 2 h to reach the adsorption equilibrium; and (2) the changes in the acetaldehyde and carbon dioxide concentrations were evaluated by gas chromatography (GC-2014, Schimatzu) for the next 12 h. Because the samples were active only on UV spectra, irradiation was also performed by a UV source (270–400 nm, 0.08 mW/cm^2^).

## Results and Discussions

### Crystallinity and Morphology

The crystalline structure was evaluated in order to observe if the thermal treatment and the additives induced changes in the tandem structures. Considering the most representative orientation plane for each component, the crystallite size values were calculated with the Scherrer formula (Equation 1) from the full-width-half-maximum (FWHM) values obtained from the XRD. The results are presented in Table 1.

(1) $$\begin{array}{l} D=\frac{K\cdot \lambda }{FWHM\cdot \text{cos }\theta }=\frac{0.9\cdot \lambda }{FWHM\cdot \text{cos }\theta } \end{array}$$

**Figure 1:**
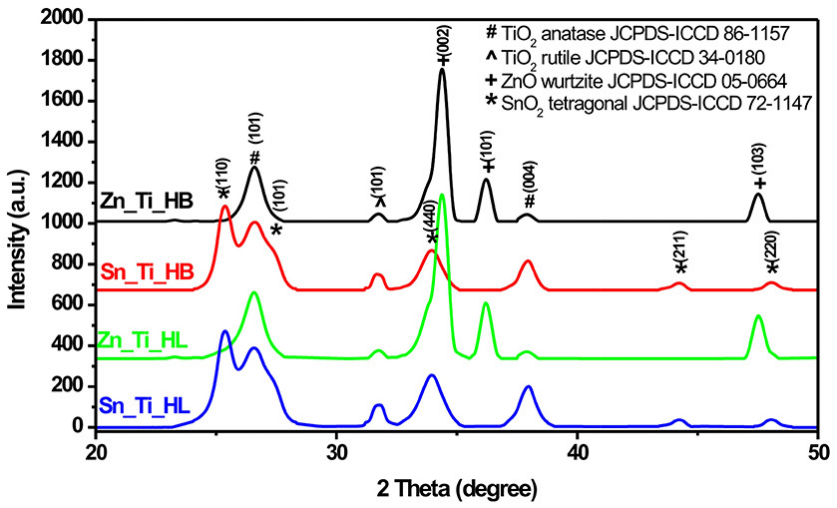
XRD patterns of the tandem structures.

Figure 1 shows the presence of anatase (ICCD 86-1157) and rutile (ICCD 34-0180) TiO_2_, which is a characteristic of Degussa powder. TiO_2_ exhibited a slight increase in crystallite sizes from an average value of 22 nm (anatase)/24 nm (rutile) in the presence of HL additive to 23 nm (anatase)/25 nm (rutile) when HB was employed as additive. The presence of the wurtzite ZnO structure (ICCD 05-0664) was identified in both Zn_Ti_HL and Zn_Ti_HB samples. Tin oxide was present in tetragonal structure (ICCD 72-1147) with similar diffraction peaks in Sn_Ti_HL and Sn_Ti_HB tandem structures. The influence of additives on crystallite sizes was observed on both SnO_2_ and ZnO where the values vary from 44.3 nm (HL) to 45.7 nm (HB) for SnO_2_ and 29.3 nm (HL) to 30.1 nm (HB) for ZnO. Even when there was not a significant crystallite size variation the results indicate a similar influence of the organic additives, regardless of the metal oxide composition or crystalline structure. The diffraction analysis shows no additional peaks, which is an indicator that the powder crystallinity was preserved after the formation of photocatalytic films using the doctor blade technique. The thermal treatment at 450°C was employed to eliminate the organic additives used to tailor the final morphology of the samples. These results are in accordance with data from other studies (Pedanekar et al., 2020; Singh et al., 2020), which reported that the organic additive does not interfere with the pre-existing crystallinity of the metal oxides, but that it can induce the formation of the carbonaceous impurities that are not identified by diffraction analysis.

**Table 1:** Crystalline parameters of the tandem samples.

**Sample**	**Crystalline parameters**			
	**Compound**	**Crystalline latice**	**Miller index**	**Crystallite size (Å)**
Sn_Ti_HL	SnO_2_	Tetragonal	(110)/(101)/ (440)/(211)/(220)	443 (101)
	TiO_2_ anatase	Tetragonal	(101)/(004)	220 (101)
	TiO_2_ rutile	Tetragonal	(101)	245 (101)
Sn_Ti_HB	SnO_2_	Tetragonal	(110)/(101)/ (440)/(211)/(220)	457 (101)
	TiO_2_ anatase	Tetragonal	(101)/(004)	232 (101)
	TiO_2_ rutile	Tetragonal	(101)	257 (101)
Zn_Ti_HL	ZnO	Hexagonal	(002)/(101)/ (103)	293 (002)
	TiO_2_ anatase	Tetragonal	(101)/(004)	218 (101)
	TiO_2_ rutile	Tetragonal	(101)	236 (101)
Zn_Ti_HB	ZnO	Hexagonal	(002)/(101)/ (103)	301 (002)
	TiO_2_ anatase	Tetragonal	(101)/(004)	224 (101)
	TiO_2_ rutile	Tetragonal	(101)	247 (101)

**Figure 2:**
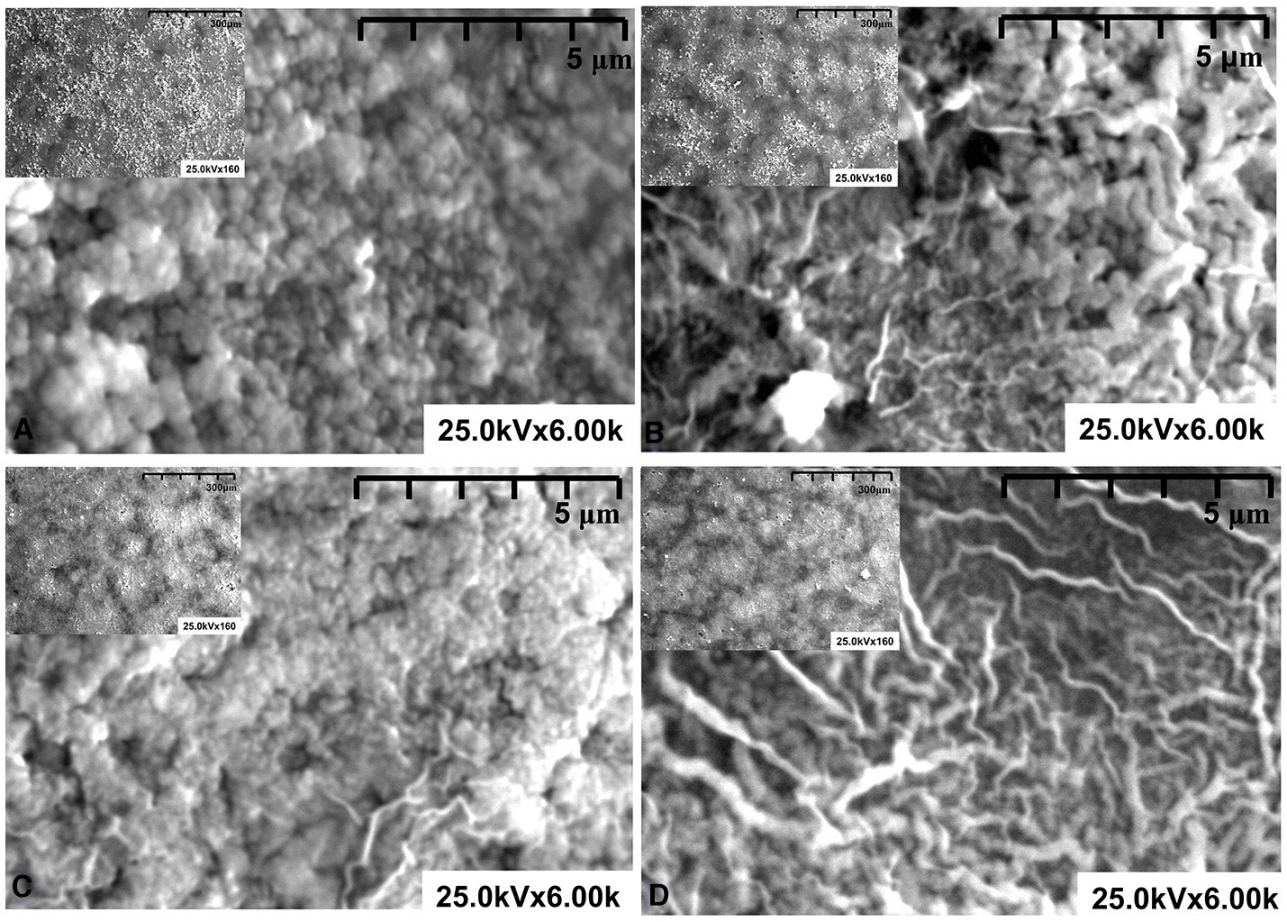
SEM pictures of: **(A)** Sn_Ti_HL, **(B)** Sn_Ti_HB, **(C)** Zn_Ti_HL, and **(D)** Zn_Ti_HB samples.

The SEM analysis (Figure 2) was performed in a semi-vacuum and no metallic covering was required for this procedure. The samples obtained using HL additive (Sn_Ti_HL and Zn_Ti_HL) present a homogeneous granular surface without defects, which is an indicator that sodium maleat-methyl metacrylate facilitates a uniform dispersion through the surface. The organic additives are known to possess the ability to self assemble (e.g., micelles, vesicles), which can serve as templates depending on the solvent composition (Wang et al., 2020; Yu et al., 2020). This behavior correlates with the macromolecular coils in this concentration range, which reduces the aggregates formed during the thermal treatment. Consequently, during the thermal treatment, the HL additive allows a uniform distribution of the metal oxide particles on the substrate surface, which explains the formation of particle-like morphology. The particles are assembled in compact structures forming dense films with thickness around 2 μm (see Table 2).

**Table 2:** Tandem structures quantitative evaluation.

**Properties**	**Sn_Ti_HL**	**Sn_Ti_HB**	**Zn_Ti_HL**	**Zn_Ti_HB**
Thickness (μm)^*^	2.14	2.93	1.93	2.24
Volume (cm^3^)	12.56 × 10^−5^	14.33 × 10^−5^	10.23 × 10^−5^	11.68 × 10^−5^
Density (g/cm^3^)	8.8	6.3	7.2	5.8
Weight (g)	11.05 × 10^−4^	9.02 × 10^−4^	7.36 × 10^−4^	6.77 × 10^−4^

* *Calculated from the reflectance spectra at 6° incident angle*.

When HB additive is used (Sn_Ti_HB and Zn_Ti_HB) the morphology change and are less uniform. Based on our previous studies (Dudita et al., 2011), sodium maleat-vinyl acetate with an average length hydrophobic tail, form micelles which exhibit electrostatic forces with the metal oxide powders, resulting in the formation of reticular structures with various lengths and diameters. During the thermal treatment, HB additives induce the formation of preferential active centers where the metal oxide particles form aggregates partially covered with reticular structures. The films are characterized by a porous morphology with a higher thickness (~2.9 μm) compared with the films obtained using HL additive. Tailoring the photocatalyst morphology is a pre-requisite in the photocatalytic applications, which is interfacing dependent.

The physical properties of the tandem structures are summarized in Table 2. The Sn_Ti_HL and Zn_Ti_HL tandem structures are characterized by lower thickness and higher density, which confirm that the HL additive enables uniform dispersion of the powder paste and the formation of compact structures. The thermal process supports the crystallization in an oxygen rich atmosphere and induces the carbon species removal, formed during the additive deposition as a result of incomplete decomposition. The formation of uniform film morphology is favored by the presence of similar crystallite sizes and structures between the tandem components. The density decreases as the film thickness increases when the HB additive is employed. In this case, the possibility of carbon species persistence into the bulk was higher, due to the behavior of the HB additive in the polymeric chain arrangement around the aggregation structures. The correspondence between morphology and density has direct consequences on the photocatalytic properties, considering that the number of active sites with high energy will influence the formation of oxidative species and the degradation of the pollutant (Augugliaro et al., 2006; Park, 2017).

### Photocatalytic Properties

**Figure 3:**
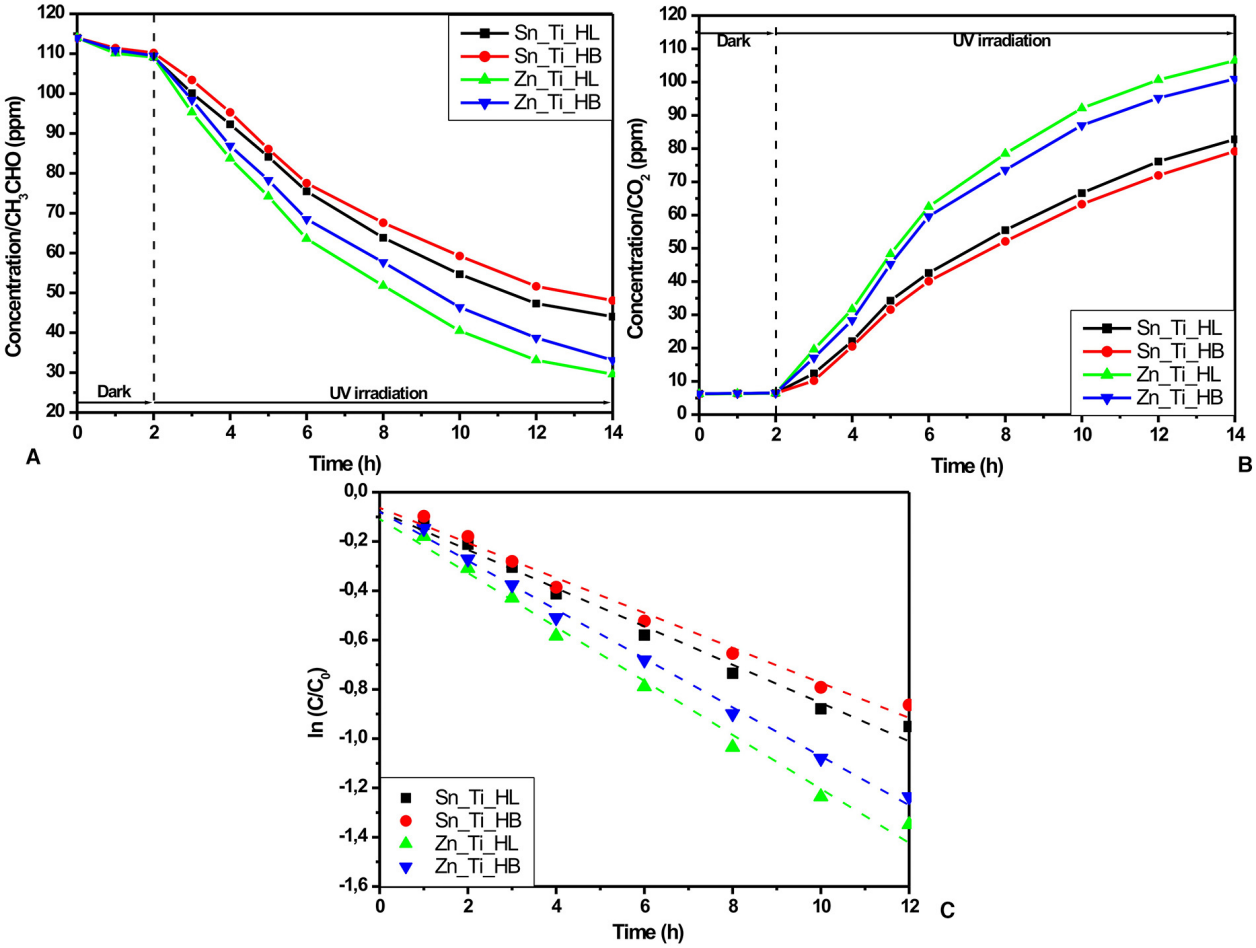
Photocatalytic activity in gaseous environment: **(A)** amount of CH_3_CHO, **(B)** amount of CO_2_, and **(C)** the kinetics of CH_3_CHO photocatalytic degradation using the Langmuir-Hinshelwood correlation.

The photocatalytic activity of the tandem heterostructures in the gaseous system was evaluated using GC measurements which allow the simultaneous investigation of both CH_3_CHO input (Figure 3A) and CO_2_ output (Figure 3B) evolution. The samples were kept in the dark for 2 h to reach the absorption-desorption equilibrium and then 12 h under UV irradiation.

The GC measurements indicate higher photocatalytic activities in the samples obtained using HL additives (Zn_Ti_HL and Sn_Ti_HL) which are characterized by granular homogenous morphology. The Zn_Ti_HL tandem structure exhibits 74.04% photocatalytic efficiency after 12 h of UV irradiation compared with 70.93%, corresponding to the Zn_Ti_HB sample. A similar difference was obtained for Sn_Ti tandem structures (61.35% for Sn_Ti_HL and 57.81% for Sn_Ti_HB). These results are a consequence of the uniform metal oxide particle distribution through the tandem structure, enabling the charge carrier to efficiently generate from both components. Additionally, the Zn_Ti tandem structures have the advantage of similar crystallite sizes, where the photo-conversion and charge transport occur simultaneously. The photocatalytic activity toward acetaldehyde was evaluated using Langmuir-Hinshelwood kinetic mechanisms (Figure 3C) based on the experimental values obtained during the 12 h of UV irradiation:

(2) $$\begin{array}{l} \text{ln }C=\text{ln }C_{0}-kt \end{array}$$

where C and C_0_ represent the initial and final concentrations, k is the rate constant and t is time. The values presented in Table 3 indicate a good correlation coefficient for all samples and a superior rate constant for ZnO-TiO_2_ tandem structures. The highest rate constant corresponds to Zn_Ti_HL (0.10953 min^−1^), while the lowest corresponds to the sample Sn_Ti_HB (0.07104 min^−1^) obtained from a precursor containing the HB additive. These results show that the Zn_Ti_HL photocatalytic activity is significantly higher and is able to remove the same amount of CH_3_CHO as Sn_Ti_HB in a shorter period (510 vs. 720 min). However, further improvements must be considered in order to increase the overall CH_3_CHO photocatalytic removal until the complete mineralization.

**Table 3:** Kinetic data corresponding to CH_3_CHO pollutant.

**Kinetic data**	**Sn_Ti_HL**	**Sn_Ti_HB**	**Zn_Ti_HL**	**Zn_Ti_HB**
Correlation coefficient *R*^2^	0.9937	0.9926	0.9951	0.9981
Rate constant k (min^−1^)	0.07771	0.07104	0.10953	0.09921

Compared with the mono-component photocatalysts reported in other papers (Adhikari et al., 2018; Khan et al., 2018; Wang et al., 2018) the ZnO-TiO_2_ and SnO_2_-TiO_2_ tandem structures present the advantages of reduced charge carrier recombination, higher charge carrier mobility, and improved charge carrier concentration, based on the simultaneous photo-conversion contribution of both semiconductors. The photocatalytic activity depends on the ability of the tandem structure to generate the oxidative species required to induce pollutant molecule mineralization, according to the following Equations (3)–(5):

(3) $$\begin{array}{l} TandemStructure+h\upsilon \to e^{-}+h^{+} \end{array}$$

(4) $$\begin{array}{l} h^{+}\left(TandemStructure\right)+H_{2}O\to •OH\left(TandemStructure\right)\\ \begin{array}{l} \;+h^{+} \end{array} \end{array}$$

(5) $$\begin{array}{l} CH_{3}CHO+•OH\to Photocatalysis\mathrm{Pr}oducts\\ \begin{array}{l} \;(e.g.:CO_{2},H_{2}O) \end{array} \end{array}$$

**Figure 4:**
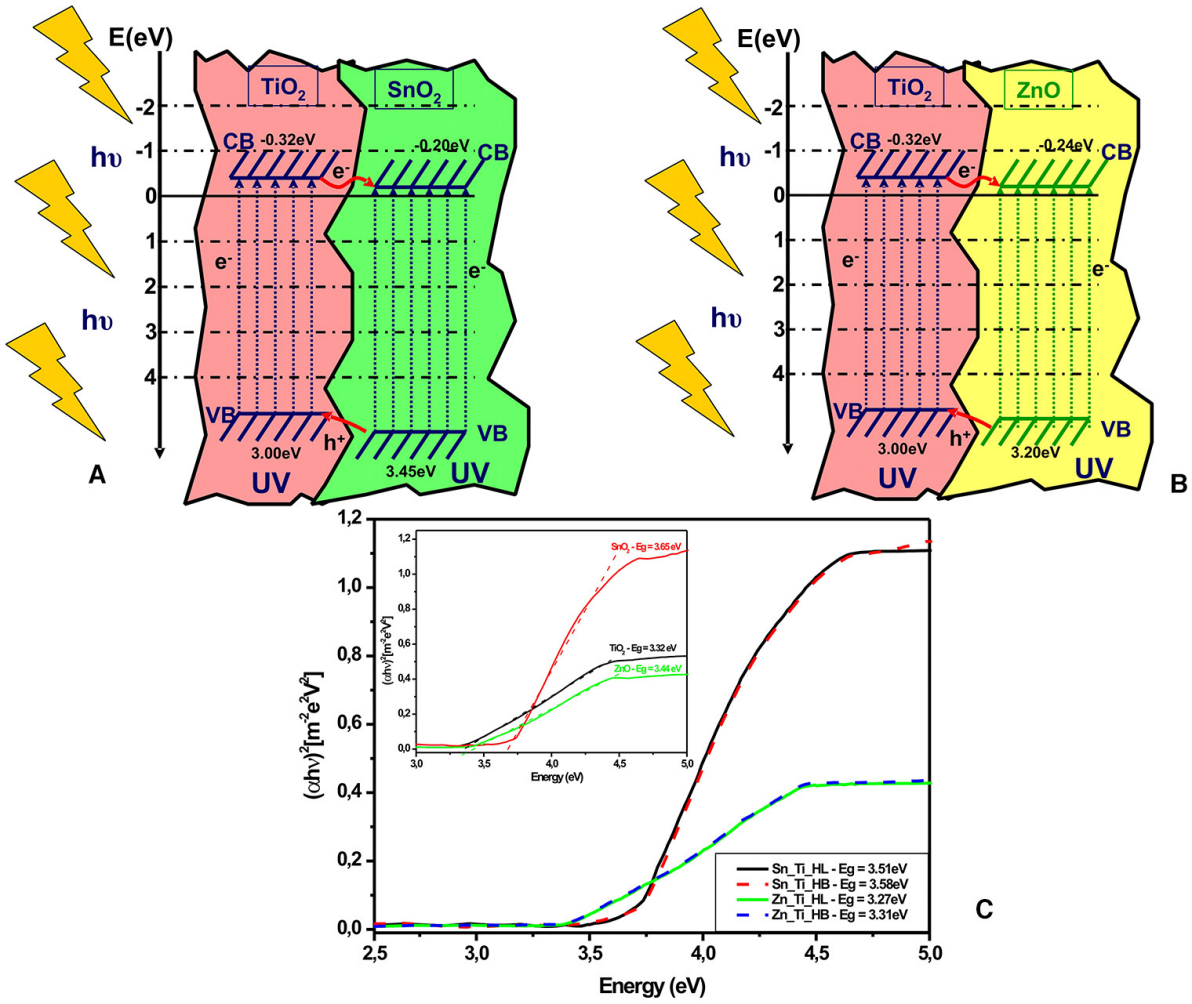
Energy levels diagrams of **(A)** Sn_Ti, **(B)** Zn_Ti, and **(C)** the tandem structures band gap evaluation (inset band gap of the single components).

The band energy diagram (Figures 4A,B) was evaluated by considering the experimental band gap values (Figure 4C) of each metal oxide, and the band energy positions were calculated by following methods presented in the literature (Gao et al., 2010; Mise and Nakada, 2010). It should be mentioned that the band gap values in tandem structures may shift but the contribution of each component of the assets is difficult considering that TiO_2_ is in a lower ratio. The procedures for evaluating the valence band (VB) and conduction band (CB) potentials of the metal oxides are based on the following equations:

(6) $$\begin{array}{l} E_{VB}=\chi_{semiconductor}-E_{e}+0.5E_{g} \end{array}$$

where EVB represents the valence band (VB) edge potential, χ_semiconductor_ represents the semiconductor electronegativity, E_e_ is the energy of free electrons vs. hydrogen, E_g_ is the band gap energy of the semiconductor (based on optical measurements), and ECB is calculated using the next equation:

(7) $$\begin{array}{l} E_{CB}=E_{VB}-E_{g} \end{array}$$

The values of absolute semiconductor electronegativity, χ_*semiconductor*_(*eV*) and the absolute cationic electronegativity, χ_*cation*_(*eV*), can be evaluated using Equations (8) and (9) where χ_*cation*_(*P*.*u*.) is the cationic electronegativity (P.u. Pauling units).

(8) $$\begin{array}{l} \chi_{semiconductor}\left(eV\right)=0.45\cdot \chi_{cation}\left(eV\right)+3.36 \end{array}$$

(9) $$\begin{array}{l} \chi_{cation}\left(eV\right)=\frac{\chi_{cation}\left(P.u\right)+0.206}{0.336} \end{array}$$

Based on the band energy diagram the electrons situated in the titanium oxide valence band are transferred on the SnO_2_/ZnO valence band representing the closest energy level. Under UV irradiation, charge carriers pairs (electron-hole) are created in the tandem structure, but only the pairs generated within the space charge region can be effectively separated by the electric field across the space charge region. The insertion of supplementary charge carriers (e^−^ and h^+^) in the depletion layer will induce a concentration gradient over the semiconductors, forming a diffuse layer. Consequently, a current flow will be formed due to a combined effect of drift and diffusion by photo-generated electrons. The conduction band (CB) edges of TiO_2_ and SnO_2_/ZnO are conveniently located at −0.32 and −0.20 (SnO_2_) or −0.24 (ZnO) eV vs. normal hydrogen electrode (NHE). The valence band (VB) edge of SnO_2_ (+3.45 eV) and ZnO (+3.20 eV) is lower than that of TiO_2_ (+3.00 eV). This mechanism is in accordance with other literature papers (Liu et al., 2012a; Badawi et al., 2013; Melhem et al., 2013) showing that a better charge separation in the tandem structures is the result of the fast electron-transfer process from the conduction band of TiO_2_ to that of SnO_2_/ZnO. The highest photocatalytic efficiency of ZnO-TiO_2_ tandem structures is related to the band energies proximity which favors the charge carrier's mobility and promotes the formation of oxidative species during the photocatalytic degradation (Riboni et al., 2013; Guo et al., 2019; Zhang et al., 2020). The influence of metal oxides ratios on the tandem structures as well as the concentration and optimization of the organic additives should be explored in future studies.

## Conclusions

In this study, four tandem structures based on ZnO-TiO_2_ and SnO_2_-TiO_2_ were prepared by the doctor blade technique using hydrophilic and hydrophobic additives. The results indicate the possibility of increasing crystallite size in the presence of an HB additive. The samples in which the HL additive was used were characterized by uniform granular morphology, high density, and lower thickness. Reticular morphology and lower density correspond to the samples where HB additives induced the formation of aggregates.

The photocatalytic activity toward acetaldehyde was tested in gaseous systems using UV irradiation and the highest efficiency (74.04%) corresponding to sample Zn_Ti_HL. The kinetics reaction corresponds to pseudo first order Langmuir-Hinshelwood, indicating a rate constant of 0.10953 min^−1^ for Zn_Ti_HL and 0.00771 min^−1^ for Sn_Ti_HL tandem structures. The tandem system based on ZnO and TiO_2_ was obtained using HL additive and exhibited superior photocatalytic activity due to the uniform particle distribution through the tandem structure as well as the similar crystallite sizes between the two metal oxides. Based on the band energy diagrams, the tandem structures can promote the mobility of charge carriers and the formation of oxidative species, which is required for pollutant degradation.

## Data Availability Statement

All datasets generated for this study are included in the article.

## Author Contributions

AE was responsible for sample preparation, data interpretation, literature review, and manuscript writing. The experimental investigation and photocatalytic studies were conducted with the support of the Tokyo University of Science.

## Conflict of Interest

The author declares that the research was conducted in the absence of any commercial or financial relationships that could be construed as a potential conflict of interest.
